# Positive impedance humidity sensors via single-component materials

**DOI:** 10.1038/srep25574

**Published:** 2016-05-06

**Authors:** Jingwen Qian, Zhijian Peng, Zhenguang Shen, Zengying Zhao, Guoliang Zhang, Xiuli Fu

**Affiliations:** 1 School of Engineering and Technology, China University of Geosciences, Beijing 100083, P. R. China; 2 State Key Laboratory of Information Photonics and Optical Communications, and School of Science, Beijing University of Posts and Telecommunications, Beijing 100876, P. R. China; 3 School of Science, China University of Geosciences, Beijing 100083, P. R. China

## Abstract

Resistivity-type humidity sensors have been investigated with great interest due to the increasing demands in industry, agriculture and daily life. To date, most of the available humidity sensors have been fabricated based on negative humidity impedance, in which the electrical resistance decreases as the humidity increases, and only several carbon composites have been reported to present positive humidity impedance. However, here we fabricate positive impedance humidity sensors only via single-component WO_3−x_ crystals. The resistance of WO_3−x_ crystal sensors in response to relative humidity could be tuned from a negative to positive one by increasing the compositional x. And it was revealed that the positive humidity impedance was driven by the defects of oxygen vacancy. This result will extend the application field of humidity sensors, because the positive humidity impedance sensors would be more energy-efficient, easier to be miniaturized and electrically safer than their negative counterparts for their lower operation voltages. And we believe that constructing vacancies in semiconducting materials is a universal way to fabricate positive impedance humidity sensors.

Resistivity-type humidity sensors, which can perceive and record the change in electrical resistance in response to that in environmental humidity, have been investigated with great interest due to the increasing demands in industry, agriculture and daily life^1^,^2^,^3^,^4^,^5^. To date, most of the available humidity sensors have been fabricated based on negative humidity impedance, in which the electrical resistance decreases as the humidity increases. However, due to their lower operation voltages, positive humidity impedance sensors would be more energy-efficient, easier to be miniaturized and electrically safer than their negative counterparts. Thus they would have wider applications in protectors for integrated circuits from humidity, energy-efficient automatic air humidifiers, and so on. But so far only several carbon composites have been reported to present positive humidity impedance^6^,^7^,^8^.

In sensing materials, semiconductor metal oxides are one of the most promising candidates for solid-state chemical sensors due to their high sensitivity, and quick response and recovery^9^,^10^. Among them, tungsten oxides are very important semiconducting materials, finding applications in gas sensing together with photocatalysis and electrochromism^11^. Focusing on gas sensors, tungsten oxides can be applied for a variety of gases, such as H_2_S, O_2_, NO_x_, CO_x_, NH_3_ and so on^12^,^13^,^14^. Particularly, the sensors for H_2_O (humidity) based on WO_3_ (the only reported tungsten oxide based sensors in literature) are WO_3_ nanowire humidity sensor on chip manufactured using CMOS-MEMS technique^15^ and WO_3_ thin-film sensor fabricated using deposition technology^16^. But in most cases, they also functionalize in a composite, just like poly-2,5-dimethoxyaniline/WO_3_ composites^17^, the mixture of Cr_2_O_3_ and WO_3_^18^, and polyaniline/WO_3_ composites^19^. And none of them exhibits positive-sensitive property to himidity. As for the sensing mechanism, the response of WO_3_ to relative humidity (RH) is generally attributed to the water dissociative chemisorptions process that would result in the formation of hydroxyl groups on the surface of WO_3_ crystals; and then, electrons are accumulated on the WO_3_ surface. As a result, the resistance of WO_3_ crystals decreases with increasing RH^17^,^20^. To the best of our knowledge, no study focuses on the influence of oxygen vacancies density of metal oxides on humidity sensing property.

Furthermore, unlike most of the oxygen-deficient metal oxides, which are not stable (especially in humid condition), WO_3−x_ crystals with a variety of oxygen-deficient stoichiometries, such as WO_2.72_, WO_2.8_, WO_2.83_ and WO_2.9_, can be easily prepared, since they are stable, ordered phases with precise stoichiometries. And the early studies revealed that oxygen vacancy can consistently account for the defect level and trap assisted conduction in semiconducting oxides^21^,^22^,^23^,^24^. Among them, Gillet and co-workers^24^ even indicated that the density of oxygen vacancy in WO_3_ would be affected by water vapor when the experiments were performed in air. These facts inspire us to design and fabricate various WO_3−x_ humidity resistors in which the different densities of oxygen vacancy might induce and modulate the humidity sensitivity.

Therefore, here we developed an approach to prepare oxygen-deficient tungsten oxides (WO_3−x_) nano-/micron-structures (NMS) only by heating WO_3_ powder in S atomsphere in a vacuum tube furnace, and with the structured WO_3−x_ crystals, humidity sensors were fabricated simply by screen-printing them onto ceramic substrates with Ag-Pd interdigital electrodes. Surprisingly, a positive humidity-sensitive property was found in the sensors prepared by single-component WO_3−x_ crystals with high density of oxygen vacancies. And the resistance of WO_3−x_ crystal sensors in response to relative humidity could be tuned from a negative to positive one by increasing the compositional x. We believe that our method not only provides a new avenue for fabricating highly effective positive humidity sensors by various metal oxides, but also creates a powerful platform to understand and design desirable semiconducting oxides humidity sensors. In addition, the findings on the positive resistance characteristics of single-component material humidity sensors can not only extend the application of humidity-sensitive resistor in different types of miniaturized devices, but also enrich and compensate for the humidity-sensing principles.

## Materials composition and structure

**Figure 1:**
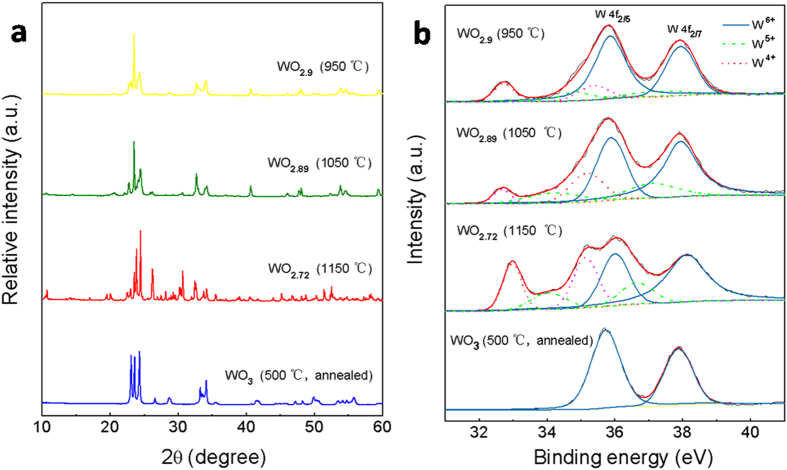
XRD (**a**) and XPS (**b**) patterns of the samples. The WO_2.9_, WO_2.89_ and WO_2.72_ crystals were synthesized by thermal evaporation of WO_3_ powder in S atomsphere at the selected temperature of 950, 1050 and 1150 °C, respectively. The WO_3_ crystals were synthesized at 1150 °C but further annealed at 500 °C in air for 2 h in a muffle furnace.

After a systematical investigation^25^, NMS samples (see Extended Data Fig. 1) with different compositions could be obtained. The phase structure of the samples was investigated by X-ray diffraction (XRD). Typical XRD patterns are shown in Fig. 1a. All the diffraction peaks of the sample prepared at 950 °C can be indexed to those of the already known monoclinic W_10_O_29_ phase (WO_2.9_, JCPDS card no. 05-0386). The XRD pattern of the sample prepared at 1050 °C matches well with that of the monoclinic W_19_O_55_ (WO_2.89_, JCPDS card no. 45-0167). And the sample synthesized at 1150 °C consists of monoclinic W_18_O_49_ (WO_2.72_, JCPDS card no. 05-0392). However, the diffraction peaks of their corresponding annealed samples are all well assigned to those of the identical phase, monoclinic WO_3_ (JCPDS card no. 43-1035).

To determine the chemical state of the elements in the obtained samples, X-ray photoelectron spectroscopy (XPS) analysis was carried out. The results are shown in Fig. 1b. For tungsten, a complex energy distribution of W4f photoelectrons was obtained. The W4f core-level spectrum could be deconvoluted into three doublets (six peaks), which are also shown in this figure, where the red line corresponds to the fitted spectrum. The binding energies of the first doublet peaks (solid blue curve) are 35.85 and 37.9 eV for W4f_7/2_ and W4f_5/2_ lines, respectively, which can be assigned to those of the W^6+^ oxidation state^26^. The second doublet peaks (dash dot green curve) have binding energies at 34.1 and 37.1 eV, corresponding to W4f_7/2_ and W4f_5/2_ lines, respectively, which can be attributed to those of the W^5+^ oxidation state^26^. The last doublet peaks (dot rose red curve consist of W4f_7/2_ line at 32.7 eV and W4f_5/2_ at 35.2 eV, indicating the existence of W^4+^ oxidation state on the sample surface^27^. The presence of three oxidation states for W ions reveals that the as-synthesized NMS are all of oxygen-deficient stoichiometries, and from the area ratio of W^6+^ over W^5+^ and W^4+^ in the spectra, it was calculated that the formula of the three samples are WO_2.9_ (synthesized at 950 °C), WO_2.89_ (at 1050 °C) and WO_2.72_ (at 1150 °C), respectively, in which the mean valence of their W ions decreased from 5.58 to 5.27 (see Extended Data Table S1). In addition, all the annealed samples were completely oxidized, containing only W^6+^ atoms, without any W^5+^ or W^4+^ atoms. And all these results are well consistent with those from XRD analysis. It should be noted that because all the annealed samples present the same XRD and XPS results, so in the discussion about WO_3_ samples, we just choose one typical annealed sample for comparison.

**Figure 2:**
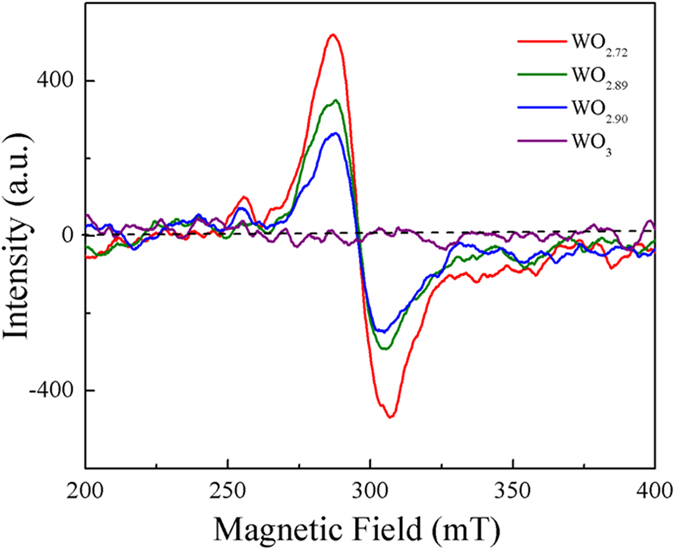
ESR spectra of the obtained tungsten oxides samples.

The electron spinning resonance (ESR) spectra of the as-prepared tungsten oxides NMS recorded at room temperature are displayed in Fig. 2. From this figure, it could be easily found that the ESR spectra of the oxygen-deficient WO_3−x_ crystals exhibit a sharp signal at *g* =  2.28, while that of WO_3_ presents no obvious signals. The signal of the present oxygen-deficient WO_3−x_ compounds could be attributed to oxygen vacancies. In literature, it was reported that the peak assigned to oxygen vacancies in metal oxides always has a *g* factor of 2.01^28^,^29^,^30^,^31^, and most of the excessive electrons are localized at the oxygen vacancies sites. Usually, one oxygen vacancy bounds one extra electron. However, the situation in oxygen-deficient WO_3−x_ compounds is more complex. Di Valentin and Pacchioni^32^ ever analyzed experimentally and computationally the spectroscopic data of WO_3−x_, indicating that sometimes the oxidation state of the under-coordinated W ions is still formally of a chemical valence of + 6, while two extra electrons are trapped in the vacancy voids, which can be explained by the following synthetic expression: W^6+^/V_O_(2e^−^)/W^6+^ (V_O_ is the oxygen vacancy). Thus, the shift of the *g* value for the present oxygen-deficient WO_3−x_ compounds to a higher one might be owing to the presence of two charge centers (two unpaired electrons) trapped in the oxygen vacancy. Moreover, the sharp *g* signal of metal oxides becomes stronger with increasing concentration of oxygen vacancies^28^,^29^,^30^,^31^. For the present oxygen-deficient WO_3−x_ compounds, as the value of x increased, the sharp *g* signal gets stronger and stronger, indicating that the density of oxygen vacancies in them increases. But there is no signal in the ESR spectrum of WO_3_, implying that the annealed samples were almost completely oxidized, containing very little or even no oxygen vacancies. This result is also in good agreement with the XPS spectra.

### Humidity sensitivity of WO_3−x_ sensors

**Figure 3:**
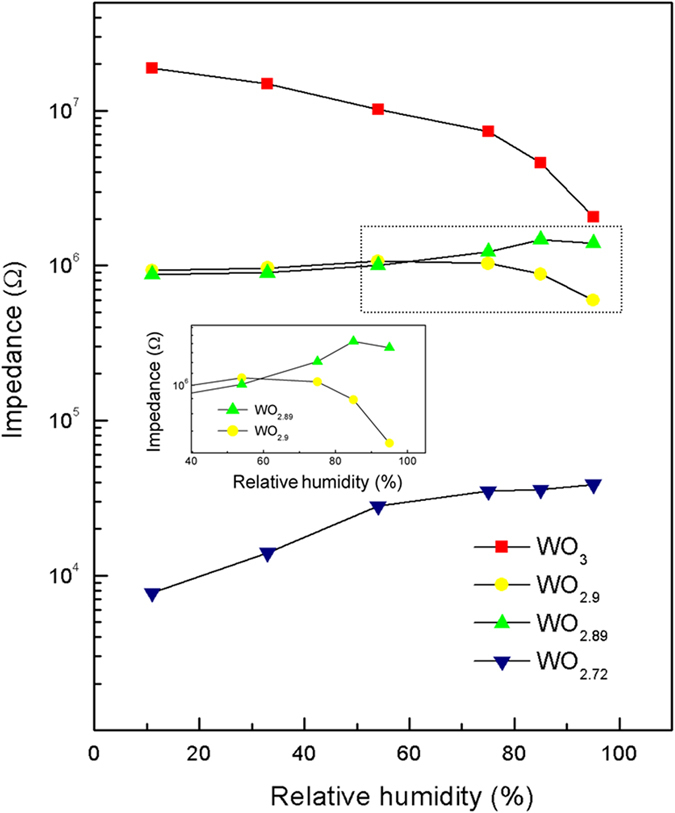
Impedance vs. relative humidity of the sensors fabricated with the obtained WO_3−x_ crystals.

The dependence of impedance on RH was measured for the sensors fabricated with the obtained WO_3−x_ crystals (see Fig. 3), in which all the dried sensing WO_3−x_ films were of about 171 μ m in thickness. From the curves, it can be clearly seen that for all the four kinds of samples, under low-humidity environment (here 11% RH), the sensors fabricated with WO_2.72_ present the lowest impedance, and the impedance of the sensors fabricated with WO_2.89_ is lower than that with WO_2.9_, while the resistance of the sensors with WO_3_ is the highest. That is to say, under low RH environment, the higher the density of oxygen vacancies in tungsten oxide crystals, the lower the impedance of the senores fabricated with them, which is similar with the conduction behavior of many semiconducting oxides^22^. The reason for this phenomenon is that oxygen vacancies are the centers of positive charges, which bound electrons easily, and the electrons around oxygen vacancies are easily excited to the conduction band; thus the conductivity of semiconducting materials can be improved with increasing density of oxygen vacancies.

Moreover, from Fig. 3, it can also be seen that the resistance of the sensors fabricated with WO_2.72_ NMS increases remarkably at low humidity (11~75% RH), and still increases somewhat at higher humidity (75~95% RH), presenting a completely positive resistance sensitivity to RH. With decreasing density of oxygen vacancies in tungsten oxide crystals, however, the fabricated sensors will present different sensing behaviors. The sensors fabricated with the as-prepared WO_2.89_ nanorods exhibits a positive resistance response to humidity in the range of 11–85% RH with slightly increased resistance. But at the humidity higher than approximately 85% RH, a negative response could be observed in such sensors, presenting an extreme point there (see the inset of Fig. 3). When the density of oxygen vacancies was further reduced (here in the WO_2.9_ nanorods), the response curve recorded from the sensors still exhibits a similar sensing profile with that of WO_2.89_ sensors, but the extreme point was found at a lower humidity of about 54% RH. When it comes to WO_3_ NMS, with increasing RH, the impedance of the sensors fabricated with the annealed NMS rapidly decreased monotonously, presenting a completely negative resistance characteristic to RH, which is in accordance with the already reported humidity sensitivity of the sensors fabricated with other forms of WO_3_ materials^15^,^16^ (also see Extended Data Fig. S2). In summary, the humidity sensitivity of the sensors fabricated with the present structured tungsten oxide crystals will display a gradual transition from a positive humidity-sensitive property to negative one depending on the density of oxygen vacancies in the sensing materials. When the density of oxygen vacancies is high (in WO_3−x_ samples with x >  0.11 in this study), they will present a completely positive humidity-sensitivity in the entire RH range (here from 11–99%). With a medium x (0.1 ≤  x ≤  0.11 in this work), they will exhibit a positive humidity-sensitive property at low RH, but still a negative one at high RH. In such case, the extreme point may gradually move to a lower RH with decreasing x. When the density of oxygen vacancies is low (in samples with x <  0.1), for example with x =  0 as in the present WO_3_ NMS, the fabricated sensors may show an almost completely negative humidity sensitive characteristics in the entire RH range.

**Figure 4:**
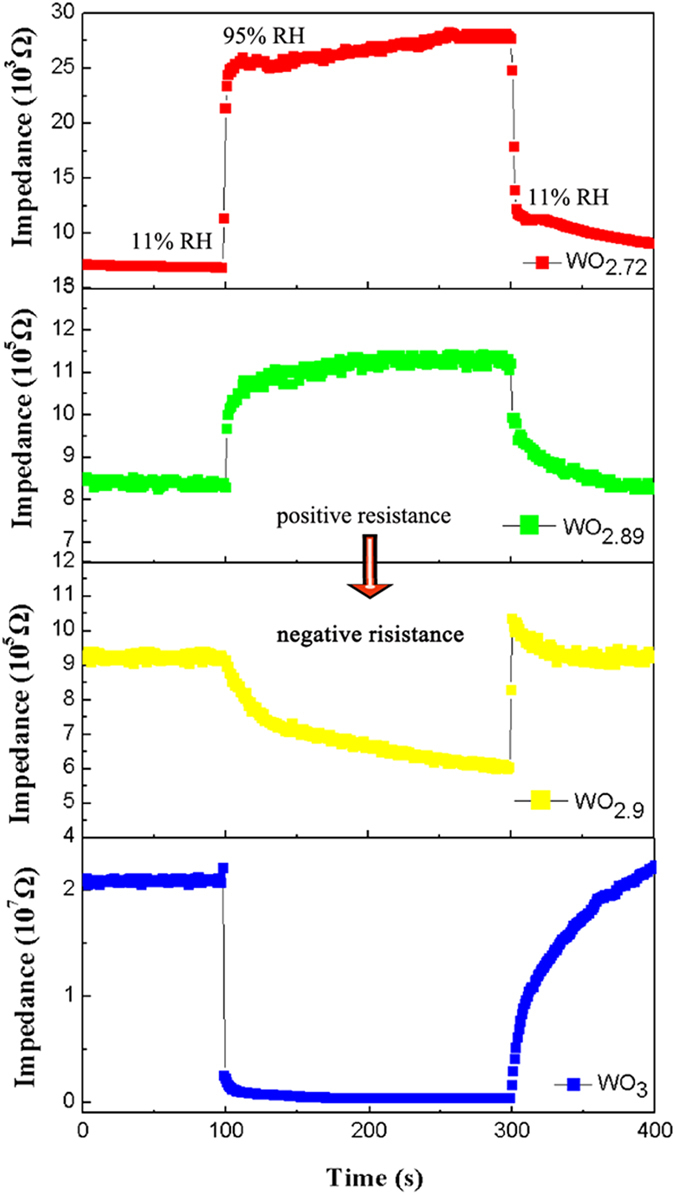
Response and recovery characteristic curves of the sensors fabricated with the obtained WO_3−x_ crystals.

It is well known that the response and recovery behavior is an important characteristic for evaluating the performance of humidity sensors. Figure 4 shows the response and recovery characteristic curves for one cycle (corresponding to the adsorption and desorption processes of water molecules) of the sensors fabricated with the obtained tungsten oxide crystals. It can be seen that, as the humidity increased from 11–95% RH, the impedance of the sensors with WO_2.72_ and WO_2.89_ increased, showing a positive resistance characteristic, but the humidity-sensitive resistors fabricated with WO_2.9_ and WO_3_ presented a negative humidity impedance characteristics, i.e., the impedance of the sensors decreased as the humidity increased. For the sensors with WO_2.72_, the impedance increased from 6.9–26 kΩ, presenting a gain of 276.8% as the humidity increased from 11–95% RH. As the density of oxygen vacancies decreased, the impedance gain of the fabricated sensors would decrease. For example, the sensors with WO_2.89_ still had a gain of about 33% with the impedance from 837–1114 kΩ. But when it comes to WO_2.9_, the impedance of the as-fabricated sensors decreased from 936–627 kΩ under similar RH environment. And the resistance of the sensors with WO_3_ even presented a sharp decrease from 20716–424 kΩ (also see Extended Data Fig. 3). Such phenomenon is correspondent with the results as shown in Fig. 3.

**Figure 5:**
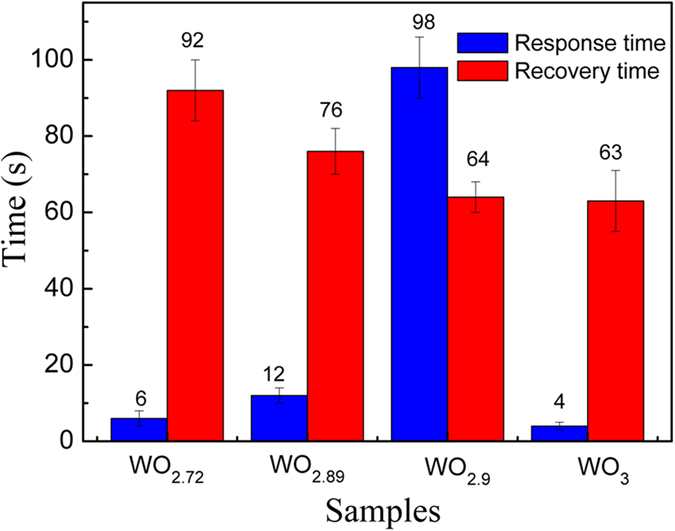
Statistical response and recovery times of the sensors fabricated with the obtained WO_3−x_ crystals.

From the response and recovery characteristic curves as shown in Fig. 4, the response time of the sensors with the obtained WO_3−x_ crystals (defined by that a sensor reaches 90% of the total impedance change as the humidity increases from 11–95% RH) and their recovery time (defined by that a sensor reaches 90% of the total impedance change as the humidity decreases in the opposite direction and range) can be calculated. Figure 5 shows their statistical response and recovery times. It can be seen that, due to the different densities of oxygen vacancies in the sensing WO_3−x_ crystals, both of the response and recovery times changed dramatically as the environment humidity varied. The response time of the sensors with WO_2.72_ NMS was about 6 s, indicating that such sensors have a very good response to humidity, which is comparable with those of the well-known WO_3_ based sensors^17^,^18^,^19^. The sensors with the present WO_3_ NMS also displayed a smiliar, quick response time of 4 s in the humidification process. But for the sensors with WO_2.89_ and WO_2.9_ NMS, they should take a longer response time (approximately 12 and 98 s, respectively) in the humidification process. The sensors with WO_2.72_ or WO_3_ NMS would display a quick response time, because both of them simply present a completely positive or negative humidity sensitive characteristics in the entire RH range (11–99% RH). However, because the sensors with WO_2.89_ and WO_2.9_ NMS exhibit a positive humidity-sensitive property at low RH but still a negative one at high RH, their response times become longer. This phenomenon might be correlated with the strong competition between the oxygen vacancies induced positive humidity sensibility and water-related negative humidity sensitive property. As for the recovery time of the sensors fabricated with the obtained WO_3−x_ crystals, as shown from the Fig. 5, it would be reduced as the density of oxygen vacancies decreased, which could be understood from the fact that the desorption kinetic energy of water from the surface of WO_3_ is generally smaller than the one of hydroxyl groups from the oxygen vacancies^33^,^34^.

In addition, it may be worth noting that the response curve of WO_2.9_ (yellow in Fig. 4) is very special and different from those of the other sensors. When the RH decreases from 99–11%, it will pass through the extreme point of the curve, where the humidity-sensitive property changes from a positive to negative one. In such case, the response curve will increase initially, and then decrease, as shown in Fig. 4. This phenomenon is correlated with the conduction mechanism of WO_2.9_, in which oxygen vacancies induced conduction dominate at low RH, but water-related electrolytic conduction at high RH.

**Figure 6:**
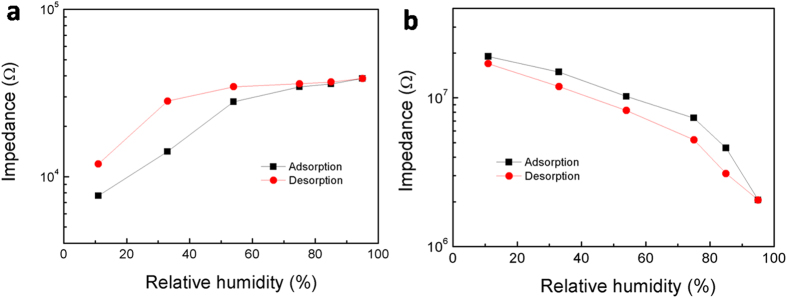
Typical humidity hysteresis of the tungsten oxides sensors. (**a**,**b**) Samples fabricated with the as-synthesized WO_2.72_ NMS and their corresponding annealed WO_3_ counterparts.

Figure 6 presents typical humidity hysteresis of the sensors fabricated with WO_2.72_ and WO_3_ NMS (also see Extended Data Fig. 4), respectively. The black lines in this figure were measured from low to high RH (for the adsorption process), and the red lines were done in the opposite direction (for the desorption process). In accordance with the response and recovery characteristics, the impedance of the sensors fabricated with WO_2.72_ increased as the humidity increased, and the pathway of its desorption process was located at the higher position of the loop, revealing that the rate of the desiccation of the adsorbed water was slower than that of the adsorption. On contrary, besides the expected negative humidity impedance characteristic, the desorption process of the WO_3_ sensors was located at the lower position of the loop, which is opposite to that of WO_2.72_ sensors. Moreover, it was calculated that the largest humidity hysteresis of the sensors fabricated with WO_2.72_ and WO_3_ were about 45.8% at 33% RH and 16.7% at 75% RH, respectively. The sensors with WO_2.72_ exhibited a relatively wide hysteresis loop, indicating a somewhat slower desorption process, which is consistent with the results on their recovery characteristics as shown in Fig. 4.

A possible qualitative mechanism to explain the humidity sensing properties of the present structured tungsten oxide crystals is proposed hereafter. Due to the totally different humidity impedance characteristics displayed by these sensors, their humidity mechanisms may have a distinctive difference. For the WO_3_ sensors, due to the very little of oxygen vacancies existed in the sensing materials, the large increase in conductivity with increasing RH can be assigned to the adsorption of water molecules on the surface of the WO_3_ crystals, because the water-related electrolytic conduction mainly functionalizes as a surface mechanism^35^. At low humidity, only very few water molecules are adsorbed, so the coverage of water molecules on the surface of the WO_3_ crystals is, most probably, not continuous, based on which, the electrolytic conduction is difficult to act; thus the conductivity of WO_3_ sensor was poor. At high humidity, one or several layers of water molecules might be formed onto the surface of WO_3_ crystals; thus, the electrolytic conduction takes place along with the weak protonic transport, and even becomes dominating in the transport-process^3^. In a word, the impedance of the sensors fabricated with WO_3_ decreases greatly as the humidity increases.

On the other hand, for the sensors with the present structured oxygen-deficient WO_3−x_ crystals, the conducting mechanism due to the oxygen vacancies induced conduction would compete with the water-related surface mechanism, and even become dominating in the transport process at low humidity. In literature, the frequently observed semiconducting oxide conductivity has been often attributed to oxygen vacancies. It was reported that, one oxygen vacancy could bound two electrons as free carriers^36^,^37^ and oxygen vacancies could introduce donor levels between the conduction and valence bands^24^,^38^, which would result in increased conductivity for semiconducting oxides. At low humidity, the electrons related to the ionization of donor centers (oxygen vacancies) of the present WO_3−x_ crystals are the effective charge carriers, thus enhancing the conductivity of WO_3−x_ crystals when the density of oxygen vacancies increases. With increasing RH, water molecules are adsorbed in oxygen vacancies. The adsorbed water is easy to dissociate from oxygen vacancies, being converted into two bridging hydroxyl groups per initial vacancy via proton transfer to a neighboring bridging oxygen atom^33^,^34^. Then the number of electrons bounded in the oxygen vacancies decreases, reducing the conductivity of WO_3−x_. At high humidity, one or several layers of water molecules are formed on the WO_3−x_ crystal surface, so the water-related electrolytic conduction would happen to a large extent. Then the oxygen vacancies induced conduction and water-related electrolytic conduction would compete with each other. When x >  0.11, the oxygen vacancies induced electrical conduction is the dominant mechanism in the sensing materials, so their sensors exhibit a positive humidity impedance characteristic in the entire RH range from 11 to 99%, due to the decreased number of oxygen vacancies induced electrons with increasing RH. In such case, under high level of humidity environment, due to the competition of water-related electrolytic conduction, the rate of impedance increase of the sensors may slow down to some extent, like the behavior of WO_2.72_ sensors as shown in Fig. 3. With decreasing x to 0.11 (in WO_2.89_ sensors), the oxygen vacancies induced electrical conduction of the sensors is the primary mechanism at low humidity (lower than 85% RH), but the water-related electrolytic conduction would become dominating at high humidity (85–95% RH in this case), thus resulting in the turning point of impedance at 85% RH. As the density of oxygen vacancies is further reduced, the turning point of the sensor impedance will move toward a lower humidity (here at 54% RH for the WO_2.9_ sensors), and even disappear, presenting a totally negative humidity impedance characteristic (as shown in the present WO_3_ sensors).

In summary, humidity sensors were successfully fabricated with WO_3−x_ crystalline NMS of different densities of oxygen vacancies. The resistance of WO_3−x_ crystal sensors in response to relative humidity could be tuned by changing the compositional x. When the density of oxygen vacancies is high (in WO_3−x_ samples with x >  0.11 in this study), they will present a completely positive impedance humidity-sensitivity in the entire RH range (here from 11–99%). With 0.1 ≤  x ≤  0.11, they will exhibit a positive impedance humidity-sensitive property at low RH, but still a negative one at high RH. In such case, the extreme point may gradually move to a lower RH with decreasing x. When the density of oxygen vacancies is low (in samples with x <  0.1), for example with x =  0 as in the present structured WO_3_ crystals, the fabricated sensors may show an almost completely negative impedance humidity sensitive characteristics in the entire RH range. Moreover, the sensors fabricated with WO_2.72_ crystals possess high sensitivity with a short response time of about 6 s, a recovery time of approximately 100 s and the largest humidity hysteresis of about 45.8% at 33% RH. The humidity sensitivity of the present tungsten oxides sensors may be controlled by the combination of oxygen vacancy induced electrical conduction and water-related electrolytic conduction.

## Methods

### Materials preparation and characterization

To fabricate the proposed tungsten oxides NMS, a high-temperature thermal evaporation process via a horizontal quartz tube furnace was used^25^. In an optimum process, 1 g commercially-bought reagent-grade WO_3_ powder (Tianjin Fuchen Chemicals, China) was loaded in a quartz boat located at the center of the furnace, while another boat with 1 g Aladdin-reagent S powder was located at the upstream from the WO_3_ powder. Before heating, the quartz tube was evacuated and flushed with Ar gas repeatedly for several times to deplete the residual gases. Then the furnace was heated up to the selected temperatures of 950, 1050 and 1150 °C with a dwelling time of 1 h. After that, the furnace was cooled down naturally to room temperature. Finally, powder-like products could be collected. For the purpose of comparison, a portion of the collected powders was annealed in air at 500 °C for 2 h in a muffle furnace.

The morphology of the obtained samples was examined by a field emission scanning electron microscope (FE-SEM, S4800, Hitachi, Japan). The chemical composition was measured by an energy-dispersive X-ray (EDX) spectroscope attached to the SEM. The phase structure and composition were identified by XRD (D/max-RB, Rigaku Corp., Japan; Cu Kα radiation, and λ  =  1.5418 Å) in continuous scanning mode with a rate of 6°/min. The chemical state of the elements in the samples was investigated by XPS (Thermo escalab 250Xi, ThermoFisher Scientific, USA; non-monochromated Mg Kα radiation, photon energy 1253.6 eV), and the results were calibrated by C1s line (binding energy, 285 eV). The unpaired electron and defect in the samples were investigated by ESR (JEOL JES-FA200, Japan) at room temperature with a microwave frequency of 9.44 GHz. Diphenylpicrylhydrazyl was used for the *g* value calibration.

### Sensors fabrication and measurement

Extended Data Fig. 5 shows schematically the sensors, in which the inset (a) shows a blank device and (b) a device coated with the sensing materials. During the fabrication, 0.2 g each of the collected powders was firstly milled and mixed with 2 mL deionized water to form a paste. Then 0.1 mL of the prepared pastes was spinning-coated by a coater (KW-4 A, China) at a rotational speed of 1000 rpm for 20 s onto an alumina ceramic substrate (Company Elitetech, China) with a size of 6 mm in length, 3 mm in width and 0.5 mm in thickness, where the screen had already been printed with two Ag-Pd interdigital electrodes of five fingers with a distance of 0.15 mm. Finally, a humidity sensor was obtained after the film was dried at ambient temperature (about 25 °C) in air for 1 h. After drying, the thickness of the sensing materials was measured by an optical microscope (BX53F, Olympus, Japan). Because the sensing film thickness also affects the performance of the sensors significantly (see Extended Data Fig. S6), all the film sensors were prepared under the same conditions excepting the presently investigated composition of the sensing WO_3−x_ materials.

The characteristic humidity sensitivity of the as-fabricated sensors, including impedance vs. relative humidity, response property and humidity hysteresis, was examined by a Keithley 2410 analyzer (USA). During the measurement, the applied operation voltage was AC 1 V and operation frequency was 1000 Hz. And the controlled humidity environment was achieved by a series of super-saturation aqueous solutions with different salts of LiCl, MgCl_2_, Mg(NO_3_)_2_, NaCl, KCl and KNO_3_, which could present a relative humidity at 25 °C of approximately 11%, 33%, 54%, 75%, 85% and 95%, respectively. In typical measurement, each sensor was soaked at 25 °C in an atmosphere of different RH levels in the six chambers with different salt solutions till it reached the adsorption-desorption equilibrium for water, and then the impedances of the sensors with the RH and time were measured, respectively.

## Additional Information

**How to cite this article**: Qian, J. *et al.* Positive impedance humidity sensors via single-component materials. *Sci. Rep.* **6**, 25574; doi: 10.1038/srep25574 (2016).

## Supplementary Material

Supplementary Information
