# Supplementary material for: Positive impedance humidity sensors via single-component materials
Source: Sci Rep. 2016 May 6;6:25574. doi: 10.1038/srep25574 (PMC4858685; doi:10.1038/srep25574)
Supplement: Supplementary Information [file srep25574-s1.pdf]

SUPPLEMENTARY INFORMATION

Positive impedance humidity sensors via single-component materials

Jingwen Qian[1,2], Zhijian Peng[1*], Zhenguang Shen[1,2], Zengying Zhao[3], Guoliang Zhang[1], Xiuli Fu[2*]

[1]School of Engineering and Technology, China University of Geosciences, Beijing 100083, PR China. Tel: 86-10-82320255; Fax: 86-10-82322624; E-mail: pengzhijian@cugb.edu.cn (Z.J.P.)

[2]State Key Laboratory of Information Photonics and Optical Communications, and School of Science, Beijing University of Posts and Telecommunications, Beijing 100876, P. R. China. Tel: 86-10-62282452; Fax: 86-10-62282054; E-mail: xiulifu@bupt.edu.cn (X.L.F.)

[3]School of Science, China University of Geosciences, Beijing 100083, PR China

We would like to thank the financial support for this work from the National Natural Science Foundation of China (grant nos. 61274015, 11274052 and 51172030), Excellent Adviser Foundation in China University of Geosciences from the Fundamental Research Funds for the Central Universities, and Fund of State Key Laboratory of Information Photonics and Optical Communications (Beijing University of Posts and Telecommunications).

Jingwen Qian, et al, Extended Data Figure 1

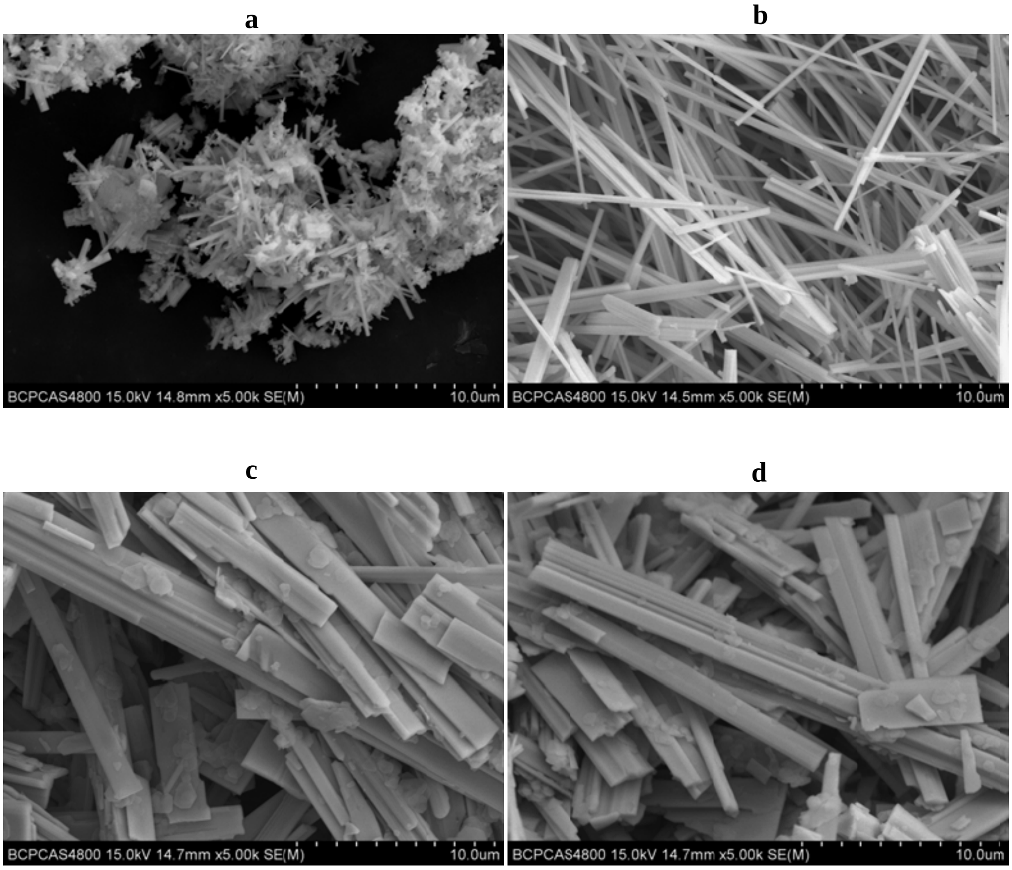

Extended Data Figure 1 | Typical SEM images of the samples. **a, b, c,** Samples synthesized by thermal evaporation of WO_3 and S powders at the selected temperature of 950, 1050 and 1150 °C, respectively. **d**, Typical annealed sample (synthesized at 1150 °C but further annealed at 500 °C in air for 2 h in a muffle furnace). For the samples synthesized at 950 °C, the SEM imaging reveals that it consists of numerous nano-rods (see Extended Data Fig. **1a**). The nano-rods are of a length of about 1-2 μm and diameter ranging from 100 to 500 nm, mixing with some particles that might be the un-reacted source powder WO_3 on the basis of morphology and SEM-EDX analyses. At 1050 °C, elongated nano-rods could be obtained (see Extended Data Fig. **1b**), which are of about 2-7 μm in length and 200-600 nm in diameter, and the source powder particles almost disappear. At 1150 °C, the resultant sample (see Extended Data Fig. **1c**) is mainly of straight nano-/micron-laths, randomly orientated in the sample, with typical length of 7-30 μm and width of 1-2 μm. For comparison, the three kinds of

as-synthesized nano-/micro-structure (NMS) were annealed respectively in oxygen atmosphere (air). Extended Data Fig. **1d** presents the SEM image of a typical sample after annealing from the obtained nano-/micron-laths as shown in Extended Data Fig. **1c**. It can be easily seen that the morphology of the annealed samples is almost the same as that of its as-synthesized counterparts, implying that during the annealing (in air at 500 ^{o}C for 2 h), the as-obtained NMS would not be damaged, destroyed or deformed, which is why we just choose one typical sample to be displayed here. In addition, SEM-EDX analysis revealed that there were only oxygen and tungsten in the as-synthesized NMS. Their O/W atomic ratios are approximately 2.11 (synthesized at 950 ^{o}C), 2.04 (1050 ^{o}C) and 1.50 (1150 ^{o}C), respectively, suggesting that they are tungsten oxides NMS and their oxygen contents decreased with increasing preparation temperature. However, after annealing under the designed conditions, the O/W atomic ratios of all the three NMS became into nearly 3:1; that is, all the annealed samples were WO_3 NMS.

Jingwen Qian, et al, Extended Data Figure 2

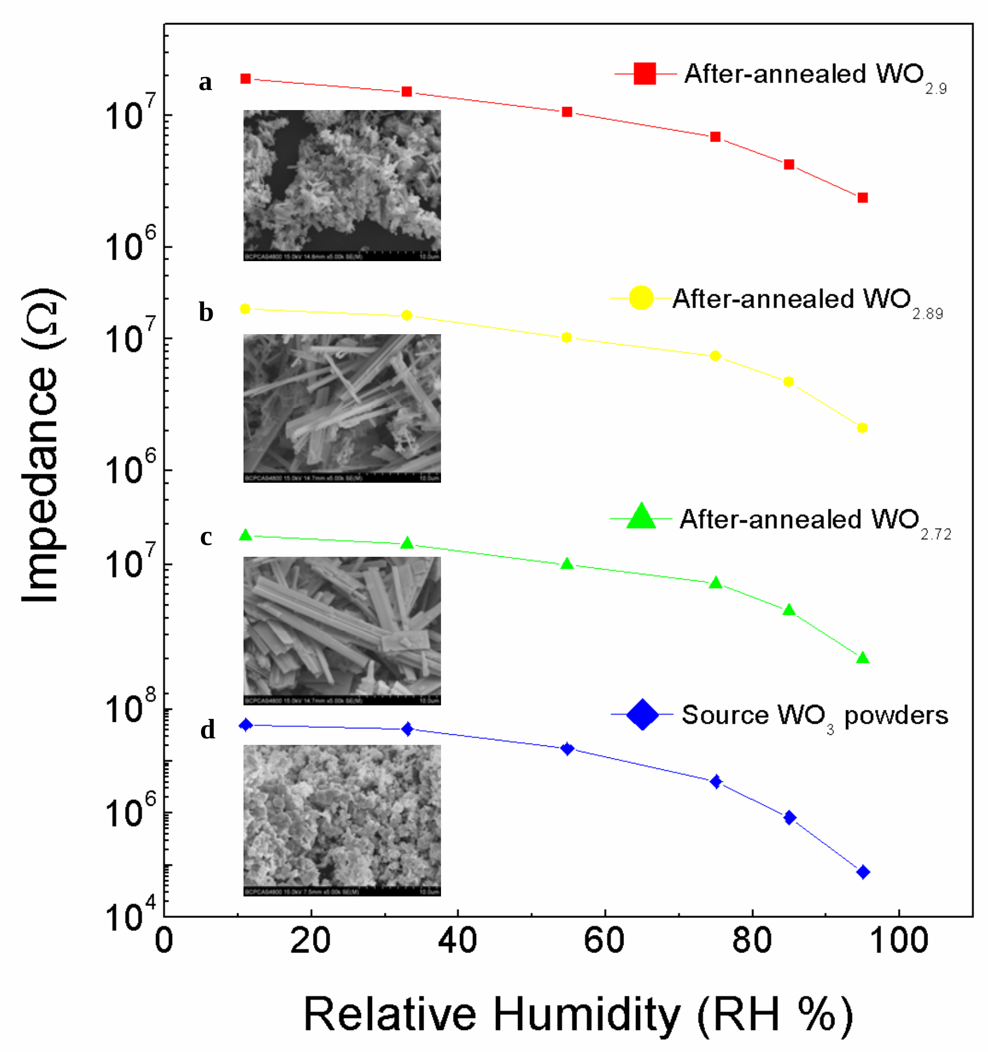

Extended Data Figure 2 | Impedance vs. relative humidity of WO_3 sensors with different morphologies. a, b, c, Sensors fabricated by NMS synthesized at the selected temperatures of 950, 1050 and 1150 °C, but further annealed at 500 °C in air for 2 h in a muffle furnace, respectively. **d,** Sensors fabricated by WO_3 source powder (with a morphology of particles). The insets display the SEM images of the NMS after annealing as well as the WO_3 source powder. This figure reveals that the sensing materials may have different morphologies, but with the same composition, the sensors fabricated by them present similar feature in impedance vs. relative humidity.

Jingwen Qian, et al, Extended Data Figure 3

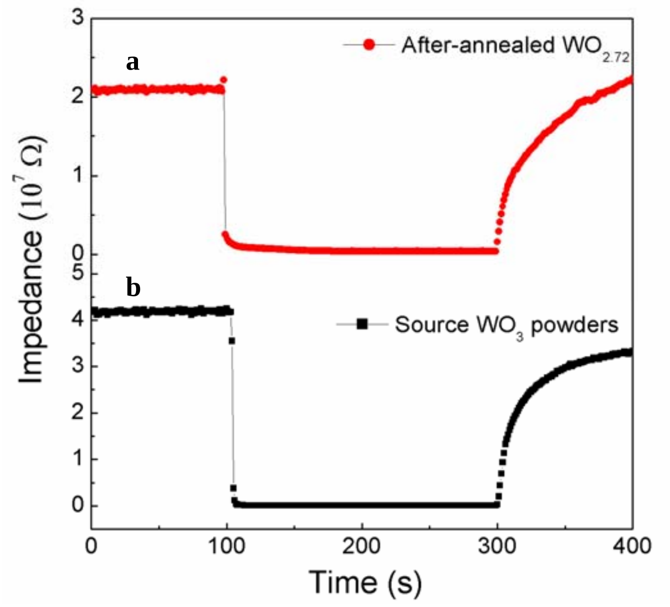

Extended Data Figure 3 | Response and recovery characteristic curves of the WO_3 sensors with different morphologies. a, Sensors fabricated by typical NMS synthesized at the selected temperatures of 1150 °C, but further annealed at 500 °C in air for 2 h in a muffle furnace. **b,** Sensors fabricated by WO_3 source powder (with a morphology of particles). This figure indicates that the sensing materials may have different morphologies, but with the same composition, the sensors fabricated by them present similar characteristics in response and recovery.

Jingwen Qian, et al, Extended Data Figure 4

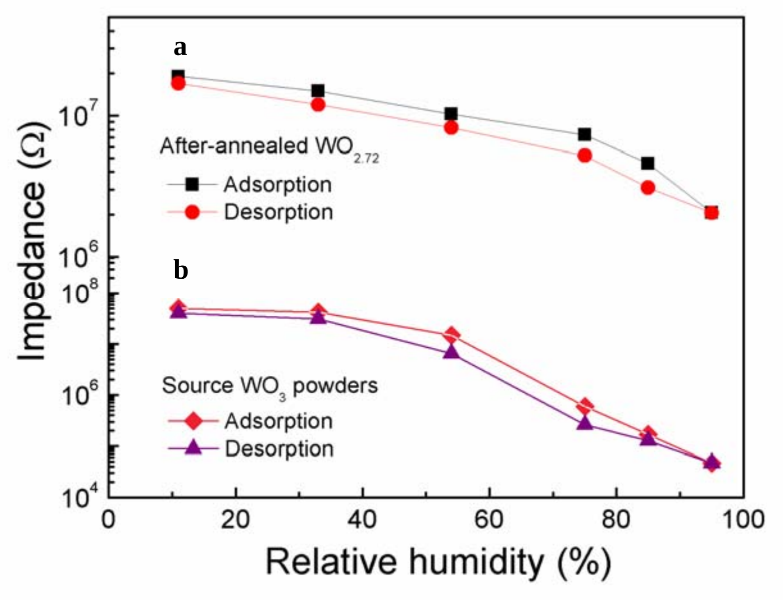

Extended Data Figure 4 | Humidity hysteresis of the WO_3 sensors with different morphologies. a, Sensors fabricated by typical NMS synthesized at the selected temperatures of 1150 °C, but further annealed at 500 °C in air for 2 h in a muffle furnace. **b,** Sensors fabricated by WO_3 source powder (with a morphology of particles). This figure displays that the sensing materials may have different morphologies, but with the same composition, the sensors fabricated by them present similar feature in humidity hysteresis.

Jingwen Qian, et al, Extended Data Figure 5

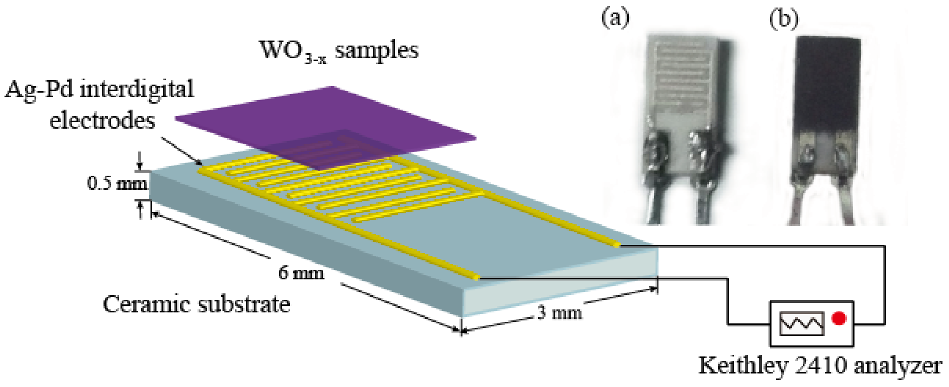

Extended Data Figure 5 | Schematic of the fabricated sensors. The inset **a** shows a blank device, and **b** a device coated with the sensing materials.

Jingwen Qian, et al, Extended Data Figure 6

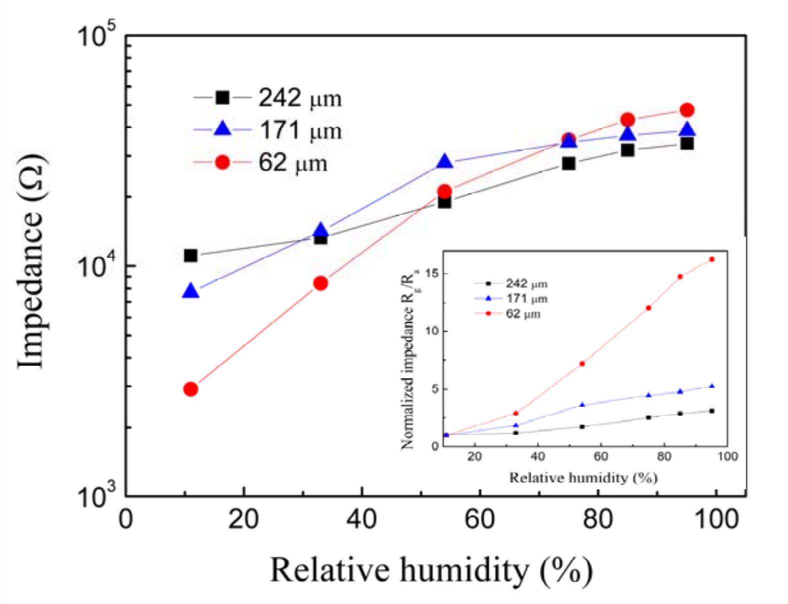

Extended Data Figure 6 | Impedance vs. relative humidity of the sensors with $WO_{2.72}$ NMS sensing films with different thicknesses, in which the inset shows the normalized resistance (R_g/R_a) vs. relative humidity. Because the thickness of sensing films depends mainly upon the spinning speed and time during coating, in order to prepare sensing films with different thicknesses, 0.1 mL of the prepared paste was spinning-coated for 20 s at a rotational speed of 500, 1000, and 2000 rpm, respectively, while all the other parameters were kept the same.

In this test, the film thickness of the three sensors was measured as about 242, 171, and 62 μm, respectively. As seen from this figure, all the sensors with different film thicknesses displayed quite good linearity, and under the dry conditions (~11% RH), the impedance (*R*) of the sensors tends to increase with increasing film thickness, which can be explained by the following equation,

$$R=\rho \cdot l/A, \quad (1)$$

in which,

$$A = t \cdot H \quad (2)$$

where ρ is the resistivity of the sensing material, l is the length between contact electrodes, A is the cross-sectional area, t is the thickness and H is the length of the sensing film[S1]. About the influence of thickness on humidity sensitivity, it could be more clearly seen from the inset of this figure, where the sensor response is expressed by normalized resistance: the resistance of the thin film devices under exposure to vapor (R_g) divided by their corresponding resistance in air of ~16% RH (R_a). The normalized resistance of the sensor with a film thickness of 62 μm at 95% RH is 16.27, much higher than those of the sensors with film thickness of 171 and 242 μm (5.26 and 3.11, respectively), and the sensitivity of the sensors always increases as the thickness decreases in the entire RH range. This result shows that a thinner film device is more sensitive to vapor than one with thicker sensing film. The difference on the sensitivity of the sensors with different film thicknesses is also correlated with their conduction mechanism. The conducting mechanism of $WO_{2.72}$ sensor in low RH is the oxygen vacancies induced conduction, and when the $WO_{2.72}$ NMS adsorbed water, the number of electrons bounded in the oxygen vacancies decreased, so the impedance would decrease. However, the water-related electrolytic conduction at high RH would reduce the impedance. And the sensors with higher sensing film thickness would present more adsorption sites of H_2O, resulting in enhanced water-related electrolyticconduction[S2]. So, at high RH, when the sensing film becomes thicker, the oxygen vacancies induced conduction would be competed by a stronger water-related surface conduction. Then the dominating oxygen vacancies induced conduction becomes weaker at high RH, and the resistance of the corresponding sensors would decrease.

S1. Almar, L. *et al.* Mesoporous ceramic oxides as humidity sensors: A case study for gadolinium-doped ceria. *Sensor. Actuat. B* **216**, 41-48 (2015).

S2. Molenda, J. & Kubik, A. Electrical properties of nonstoichiometric WO_{3-y} at temperatures 77 to 300 K. *Phys. Status Solidi B* **191**, 471-478 (1995).

Jingwen Qian, et al, Extended Data Table 1

Extended Data Table 1 | Basic information on the obtained tungsten oxides samples

Samples	Crystalline structure and morphology	Color	Size (diameter ×length, μm)[a]	Source and atmosphere	Treatment temperature (° C)	$W^{6+}/W^{5+}/W^{4+}$ (atom%)[b]	W^c	W^d
$WO_{2.9}$	Monoclinic $W_{10}O_{29}$, nano-rods	Green-blue	(0.1-0.5)×(1-2)	WO_3 and S powders, Ar	950	72.77/12.69/14.53	5.58	5.8
$WO_{2.89}$	Monoclinic $W_{19}O_{55}$, nano-rods	Dark-blue	(0.2-0.6)×(2-7)	WO_3 and S powders, Ar	1050	72.24/12.56/15.19	5.57	5.78
$WO_{2.72}$	Monoclinic $W_{19}O_{48}$, nano-/micron-laths	Purple-red	(1-2)×(7-30)	WO_3 and S powders, Ar	1150	55.29/16.77/27.94	5.27	5.44
Typical annealed samples	Monoclinic WO_3, nano-/micron-laths	Green	(1-2)×(7-30)	$WO_{2.72}$ nano-/ micron-laths, Air	500	100/0/0	6	6

a. Mean particle size determined by TEM and SEM.

b. Oxidation state of W ions determined by XPS from $W4f_{7/2}$ peaks (37.9, 37.1 and 35.2 eV for W^{6+}, W^{5+} and W^{4+}, respectively) and $W4f_{5/2}$ peaks (35.85, 34.1 and 32.7 eV for W^{6+}, W^{5+} and W^{4+}, respectively).

c. Average oxidation number counted by the oxidation state of W ions.

d. Theoretical average oxidation number.
